# The miR-361-3p increases enzalutamide (Enz) sensitivity via targeting the ARv7 and MKNK2 to better suppress the Enz-resistant prostate cancer

**DOI:** 10.1038/s41419-020-02932-w

**Published:** 2020-09-25

**Authors:** Bianjiang Liu, Yin Sun, Min Tang, Chao Liang, Chi-Ping Huang, Yuanjie Niu, Zengjun Wang, Chawnshang Chang

**Affiliations:** 1 Department of Urology, The First Affiliated Hospital of Nanjing Medical University, Nanjing, China; 2 George Whipple Lab for Cancer Research, Departments of Pathology, Urology and Radiation Oncology, and The Wilmot Cancer Institute, University of Rochester Medical Center, Rochester, NY USA; 3 Sex Hormone Research Center and Department of Urology, China Medical University/Hospital, Taichung, Taiwan

**Keywords:** Prostate cancer, Prostate cancer

## Abstract

The androgen receptor splicing variant 7 (ARv7) that lacks the ligand-binding domain is increasingly considered as a key player leading to enzalutamide (Enz) resistance in patients with prostate cancer (PCa). However, the detailed mechanisms of how ARv7 expression is regulated and whether it also needs other factors to induce maximal Enz resistance remain unclear. Here, we identified a microRNA, miR-361-3p, whose expression is lower in patients with recurrent PCa, could function via binding to the 3′UTR of ARv7, but not the wild type of AR, to suppress its expression to increase the Enz sensitivity. Importantly, we found that miR-361-3p could also bind to the 3′UTR of MAP kinase-interacting serine/threonine kinase 2 (MKNK2) to suppress its expression to further increase the Enz sensitivity. In turn, the increased Enz can then function via a feedback mechanism through altering the HIF-2α/VEGFA signaling to suppress the expression of miR-361-3p under hypoxia conditions. Preclinical studies using an in vivo mouse model with orthotopically xenografted CWR22Rv1 cells demonstrated that combining the Enz with the small molecule miR-361-3p would result in better suppression of the Enz-resistant PCa tumor progression. Together, these preclinical studies demonstrate that miR-361-3p can function via suppressing the expression of ARv7 and MKNK2 to maximally increase the Enz sensitivity, and targeting these newly identified Enz/miR-361-3p/ARv7 and/or Enz/miR-361-3p/MKNK2 signals with small molecules may help in the development of novel therapies to better suppress the CRPC in patients that already have developed the Enz resistance.

## Introduction

Prostate cancer (PCa) is the leading cause of cancer-related deaths among males in western countries^1^, and androgen-deprivation therapy (ADT) with antiandrogens remains the standard therapy to treat the castration-resistant PCa (CRPC) patients. However, most CRPC patients will eventually fail to respond to ADT, with development of antiandrogen resistance, even though the androgen receptor (AR) continues to function^2,3^.

A new high-affinity antiandrogen enzalutamide (Enz, also known as MDV3100) has been approved by the FDA, which can prolong survival in patients with metastatic CRPC, to better suppress the AR function^4–7^. However, Enz resistance eventually may also occur due to multiple mechanisms^8–11^, including recent clinical studies showing that Enz might induce the expression of some AR-splice variants^3,12–15^. The AR-splice variant 7 (ARv7) is the most studied AR variant that has clear human clinical data showing its positive linkage to the development of Enz resistance^16–19^. The detailed mechanism of how ARv7 is produced/altered and its linkage to the development of Enz resistance, however, remain unclear.

The microRNAs (miRNAs) are small noncoding RNAs (about 22 nucleotides) that can function in post-transcriptional regulation of gene expression via sequence-specific interactions with the 3′ untranslated regions (3′UTR) of mRNA targets^20,21^. Because miRNAs interact with target sequences primarily through a seed sequence, each individual miRNA can regulate different targets via this sequence^22^. Therefore, an alteration of one miRNA can be involved in physiological and pathological processes via regulating different corresponding genes. It has been shown that miRNAs may act as promoting or suppressing factors in various cancers^23^, and some selective miRNAs have been used as therapeutic agents for cancer treatment^24^.

As a result of altered RNA splicing, ARv7 has a different 3′UTR compared to the full-length AR (fAR). We hypothesize that some specific miRNAs may be able to regulate the expression of ARv7 in CRPC cells via sequence-specific interactions with its unique 3′UTR, and the consequences of such unique interactions may play key roles for the ARv7-induced Enz resistance.

Here, we identified such a miRNA, miR-361-3p, that can function via binding specifically to the 3′UTRs of ARv7 and MAP kinase-interacting serine/threonine kinase 2 (MKNK2), another tumor growth-related gene, to modulate the Enz sensitivity in CRPC.

## Materials and methods

Approval for this study was granted by the ethics committee of Nanjing Medical University, and informed written consent was received from all participants for human sample collection in our research.

### Cell culture

PCa cell lines CW22Rv1, C4-2, and LNCaP were obtained from American Type Culture Collection (ATCC, Manassas, VA, USA) and maintained in RPMI 1640 media with 10% FBS and antibiotics (100 units/mL penicillin, 100 µg/mL streptomycin). Cells were maintained in humidified 5% CO_2_ environment at 37 °C. For generation of the Enz-resistant (Enz-R) cell line, C4-2 cells were kept in media with 10 µM Enz for at least 3 months before experiments, as in our previous study^25^. Hypoxia was achieved by maintaining the cells at 1% O_2_, 5% CO_2_, and 94% N_2_ in a hypoxic chamber (Coy Laboratory Products, Grass Lake, MI, USA) with oxygen sensor controls, with temperature at 37 °C with humidity controls, and CO_2_ and N_2_ gas regulators.

### Reagents and materials

GAPDH antibody was purchased from Santa Cruz Biotechnology (Dallas, TX, USA), ARv7 antibody was purchased from Precision Antibody (Columbia, MD, USA), MKNK2 antibody from Thermo Fisher Scientific (Waltham, MA, USA), anti-mouse/rabbit secondary antibody for western blot from Thermo Fisher Scientific, and anti-Ago2 antibody was purchased from Cell Signal Technology (Danvers, MA, USA). Immunoglobulin G (IgG) antibody was purchased from Santa Cruz Biotechnology. Enz was purchased from Selleck Chemicals (Houston, TX, USA) and TC-S 7009 from Tocris Bioscience (Avonmouth, Bristol, UK).

### Lentivirus packaging

The pLVTHM-miR-361-3p, pWPI-ARv7, pLVTHM-shARv7, pLVTHM-shMKNK2, psAX2-packaging plasmid, and pMD2G envelope plasmid were transfected into 293T cells using the standard calcium chloride transfection method for 48 h to get the lentivirus supernatant, which was collected and concentrated by density-gradient centrifugation followed by immediate use or frozen at −80 °C for later use.

### RNA extraction and quantitative real-time PCR analysis

Total RNAs were isolated using Trizol reagent (Thermo Fisher Scientific), and 1 µg of total RNA subjected to reverse transcription using Superscript III transcriptase Thermo Fisher Scientific. Quantitative real-time PCR analysis was conducted using the Bio-Rad CFX96 system with SYBR green to determine mRNA expression levels of a gene of interest, which were normalized to expression of GAPDH. The miRNAs were isolated using PureLink® miRNA kit. Briefly, 50 ng of small RNAs were processed for poly A addition by adding 1 unit of polymerase with 1 mM ATP in 1 × RT buffer at 37 °C for 10 min in 10-μl volume, heat-inactivated at 95 °C for 2 min, then added 50 pmol anchor primer to 12.5 μl, and incubated at 65 °C for 5 min. For the last step of cDNA synthesis, we added 2 μl of 5× RT buffer, 2 μl of 10 mM dNTP, and 1 μl of reverse transcriptase to a total of 20 μl, and incubated at 42 °C for 1 h. Quantitative real-time PCR (QPCR) was conducted using the Bio-Rad CFX96 system with Taqman probe to determine the miRNA expression levels, which were normalized to the expression of 5S and/or U6.

### Western blot analysis

Cells were lysed in RIPA buffer, and proteins (20 µg) were separated on 10% SDS/PAGE gel and then transferred onto PVDF membranes (Millipore, Billerica, MA, USA). After blocking membranes with 5% nonfat milk, they were incubated with appropriate dilutions of specific primary antibodies (1:1000). The blots were then incubated with HRP-conjugated secondary antibodies (1:4000) and visualized using ECL system (Thermo Fisher Scientific).

### RNA immunoprecipitation

Co-immunoprecipitation (Co-IP) of microRNA ribonucleoprotein complexes with anti-Ago2 or IgG was conducted. RNA Co-IP with anti-Ago2 or IgG antibodies was extracted using TRIzol reagent (Thermo Fisher Scientific) followed by detection of mRNA level through qRT-PCR. For detecting ARv7 3′UTR interaction with miRNA-361-3p, synthetic biotin-labeled sense or antisense ARv7 3′UTR was incubated with total RNA followed by detection of miRNA-361-3p levels in the pull-down assay.

### Luciferase reporter assay

The fragments of ARv7 and MKNK2 3′UTR containing wild-type or mutant miRNA-targeting sites were constructed into psiCheck2-vector (Promega, Madison, WI, USA), named as pARv7-luc and MKNK2-luc. Cells were transfected with pLVTHM-miR-361-3p, plated in 24-well plates, and transfected with pARv7-luc and MKNK2-luc using Lipofectamine 3000 (Thermo Fisher Scientific) according to the manufacturer’s instructions. After indicated treatments, cells were lysed, and the luciferase activity was detected by the dual-luciferase Assay (Promega).

### Clonogenic formation

The 6-cm dishes were seeded with 1000 CWR22Rv1 cells per dish. Cells were transfected with miR-361-3p or miR-361-3p plus ARv7 for 2 days followed by treatment with Enz. After 1–2 weeks, cells were stained with 0.1% crystal violet, and colonies >50 cells were counted.

### Cell-proliferation assay

Cells were seeded in 96-well plates (5 × 10^3^ cells/200 μl of media per well) and cultured for 0–6 days. Cells were harvested, and viable cell numbers were calculated using 3-(4,5-dimethyl-2-thiazolyl)-2,5-diphenyl-2-H-tetrazolium bromide (MTT) agent.

### Prostate cells’ orthotopic implantation

Our animal experiments were carried out in accordance with the National Institutes of Health guide for the care and use of Laboratory animals. Male 6- to 8-week-old nude mice were purchased from NCI (Bethesda, MD, USA). The Matrigel (1:1) mixtures with 1 × 10^6^ CWR22Rv1 cells (with stable transfected luciferase) with or without lentiviral stable expression of miR-361-3p were orthotopically injected into both anterior prostates. After 1 week of implantation, the mice with vector or miR-361-3p expression were separated randomly into two groups with matching charateristics and treated with either DMSO or 35 mg/kg Enz by i.p. injection 3×/week for 2 weeks. The separation of two groups was not blinded to the operator. Tumor formation and sizes were monitored weekly during the 2 weeks of drug treatment by the noninvasive In Vivo Imaging System (IVIS). After another week and a final IVIS imaging, mice were sacrificed and tumors were removed. The tissue samples were then fixed and processed as paraffin tissue sections.

### IHC staining

Paraffin-processed sections at 5-µm thickness were mounted on poly-d-lysine-coated glass slides. Slides were dewaxed in 100% xylene and rehydrated by incubation in decreasing concentrations of alcohol, and incubated in 3% H_2_O_2_ to eliminate endogenous biotin. Briefly, sections blocked with horse serum were incubated with ARv7 antibody overnight at 4 °C. After rinsing with Tris-buffered saline, the slides were incubated for 45 min with biotin-conjugated secondary antibody (Vector Laboratories, Burlingame, CA, USA), washed, and then incubated with enzyme conjugate horseradish peroxidase (HRP)–streptavidin. Freshly prepared DAB (Vector Laboratories) was used as substrate to detect HRP, and tissues were counterstained with hematoxylin and then mounted with aqueous mounting media.

### Statistics

All statistical analyses were carried out with SPSS 16.0 (SPSS Inc., Chicago, IL, USA). The data values were presented as the mean ± SD from at least three independent experiments repeated in triplicate. Differences in mean values between two groups were analyzed by two-tailed Student’s *t* test. A *P* value < 0.05 was considered statistically significant.

## Results

### The miR-361-3p can function via binding specifically to the ARv7 3′UTR to decrease ARv7 protein expression in CRPC cells

Recent clinical data indicated that ARv7 might play a key role to induce the development of Enz resistance during ADT with Enz treatment (ADT–Enz) in the CRPC patients^18,19^. The detailed mechanisms of how ARv7 is induced or modulated, however, remain unclear.

**Fig. 1:**
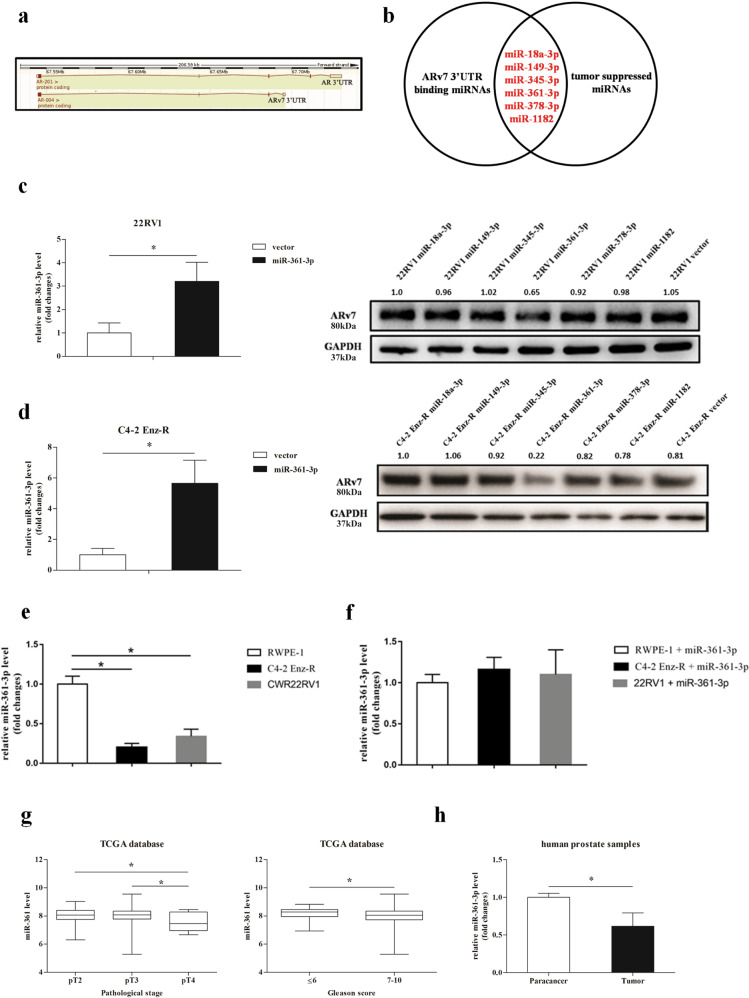
The miRNA-361-3p suppressed androgen receptor splicing variant 7 (ARv7) expression and prostate cancer (PCa) formation. **a** ARv7 has its unique 3′UTR compared to the 3′UTR of fAR. **b** The selected tumor suppressor miRNA has binding sites to the ARv7 3′UTR, and is differentially expressed in recurrent tumors. **c** Western blot analysis shows that adding miR-361-3p suppresses ARv7 protein expression in CWR22Rv1 cells. The effect of adding miRNA is shown on the left. **d** Western blot analysis shows that adding miR-361-3p suppresses ARv7 protein expression in C4-2 Enz-R cells. The effect of adding miRNA is shown on the left. **e** Expression of miR-361-3p in CWR22Rv1, C4-2 Enz-R cells, and normal prostate RWPE-1 cells. **f** Expression of miR-361-3p in CWR22Rv1, C4-2 Enz-R cells, and normal prostate RWPE-1 cells after transfecting miR-361. **g** TCGA dataset analyses indicate that miR-361-3p expression is lower in PCa with more advanced pathological stages (left) and higher Gleason scores (right). **h** Analyses of 17 PCa samples and their matched para-cancer tissues surgically collected by us indicate that miR-361-3p expression is lower in PCa. Representative results are shown as mean ± SD. *P < 0.05.

As ARv7 has a unique 3’UTR compared to the full-length AR (fAR) (see the website (http://www.ensembl.org/Homo_sapiens/Gene/Summary?g=ENSG00000169083;r=X:67544032-67730619) (Fig. 1a), we were interested to see if this unique 3′UTR in the ARv7 may contribute to its induction of the Enz resistance. We first searched online databases (TargetScan, MicroCosm, miRDB, and RegRNA) to identify a list of miRNAs that were commonly predicted to target ARv7 by different algorithms. In particular, we focused on six miRNAs, whose expression had been linked to the progression and recurrence of PCa^26–28^ (Fig. 1b). The results from the ARv7 expression study after adding these six miRNAs indicated that miR-361-3p could significantly decrease the ARv7 protein expression in both CRPC CWR22Rv1 and EnzR-C4-2 cells (Fig. 1c, d). Interestingly, the expression of miR-361-3p in PCa cells indicated higher expression of miR-361-3p in normal prostate epithelial RWPE cells versus much lower expression in CRPC CWR22Rv1 and EnzR-C4-2 CRPC cells (Fig. 1e, f).

Together, the results from Fig. 1a–f suggest that miR-361-3p can function via binding specifically to the ARv7 3′UTR to decrease ARv7 protein expression in CRPC cells.

### Human clinical survey links the expression of miR-361-3p to the PCa progression

The results from a recent human clinical survey indicated that the expression of miR-361-3p was relatively lower in PCa than in benign prostate disease^29^, and a survey from the TCGA database (http://cancergenome.nih.gov) using UCSC Cancer Genome Brower (https://genome-cancer.soe.ucsc.edu/) also revealed that the miR-361 expression in human PCa tissues is negatively correlated with pathologic TNM stage and Gleason score (Fig. 1g).

Importantly, the results from our own human clinical sample studies from 17 clinical PCa samples also confirmed the above finding showing that the expression of miR-361-3p in PCa tissues was significantly lower than those found in the normal prostate tissues (Fig. 1h).

Together, the results from various human clinical surveys (Fig. 1g, h) suggest that the expression of miR-361-3p is lower in the PCa compared to the normal prostate tissues, a strong indication that miR-361-3p may play negative roles for the PCa progression.

### Mechanism dissection of how miR-361-3p can suppress ARv7 protein expression: via direct binding to the 3′UTR of ARv7

**Fig. 2:**
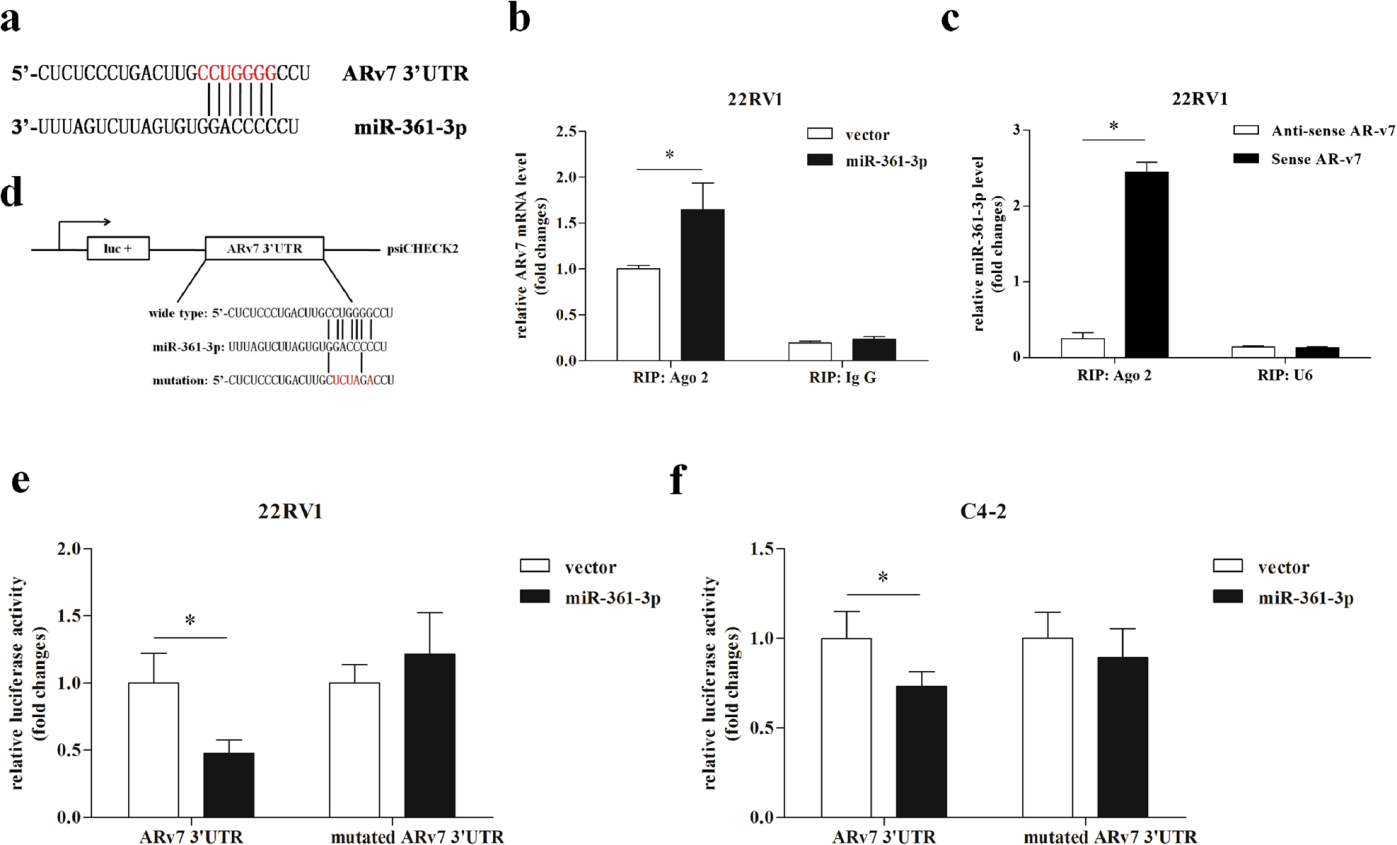
Validation of androgen receptor splicing variant 7 (ARv7) as miR-361-3p target. **a** Sequence alignment of miR-361-3p to ARv7 3′UTR. **b** RIP analysis in CWR22Rv1 cells (22Rv1) reveals recruitment of ARv7 mRNA to microRNA ribonucleoprotein complex following immunoprecipitation against Ago2. IgG immunoprecipitation is used as a negative control. **c** Synthetic sense or antisense ARv7 3′UTR with biotin is mixed with the total RNA followed by detection of miR-361-3p in the pulldown. **d** The wild-type and mutated ARv7 3′UTR via deleting the miRNA-binding sites is separately used as control for (**e**) and (**f**). **e**, **f** Luciferase reporter assay reveals that expressing miR-361-3p suppresses ARv7 3′UTR luciferase activity in 22Rv1 cells (**e**) and C4-2 cells (**f**). The assays are repeated three times with each assay being performed in triplicate wells and similar results being obtained each time. Representative results are shown as mean ± SD. *P < 0.05.

To dissect the molecular mechanism of how miR-361-3p can suppress the ARv7 protein expression, we found that miR-361-3p could bind to the 3′UTR of ARv7 (Fig. 2a). We then applied the RNA immunoprecipitation (RIP) assay using anti-Ago2 antibody for cells with lentivirus-expressing miR-361-3p to further confirm that ARv7 mRNA level increased in the miRNA ribonucleoprotein complex (Fig. 2b). To directly test that ARv7 3′UTR can interact with miR-361-3p, we synthesized the biotinylated sense and antisense strand of the 3′UTR RNA and used them as a proble to interact with total RNA from CWR22Rv1 cells followed by detection of miR-361-3p in the pull-down complex, and the results revealed that ARv7 3′UTR RNA can specifically interact with miR-361-3p, but not control U6 RNA (Fig. 2c).

Importantly, luciferase reporter assay linking ARv7 3′UTR to the luciferase cDNA (Fig. 2d) in CWR22RV1 cells also revealed that miR-361-3p suppressed ARv7 3′UTR luciferase reporter activity (Fig. 2e), and such suppression was abolished when we mutated the miRNA-target sequence in the ARv7 3′UTR in the CWR22Rv1 cells (Fig. 2d, e). Similar results were also obtained when we replaced the CWR22Rv1 cells with C4-2 cells, another CRPC cell line (Fig. 2f).

Together, the results from Fig. 2a–f suggest that miR-361-3p can suppress ARv7 protein expression via direct binding to the ARv7 3′UTR in CRPC cells.

### The consequence of the miR-361-3p-suppressed ARv7 protein expression in the CRPC cells: increasing the Enz sensitivity

**Fig. 3:**
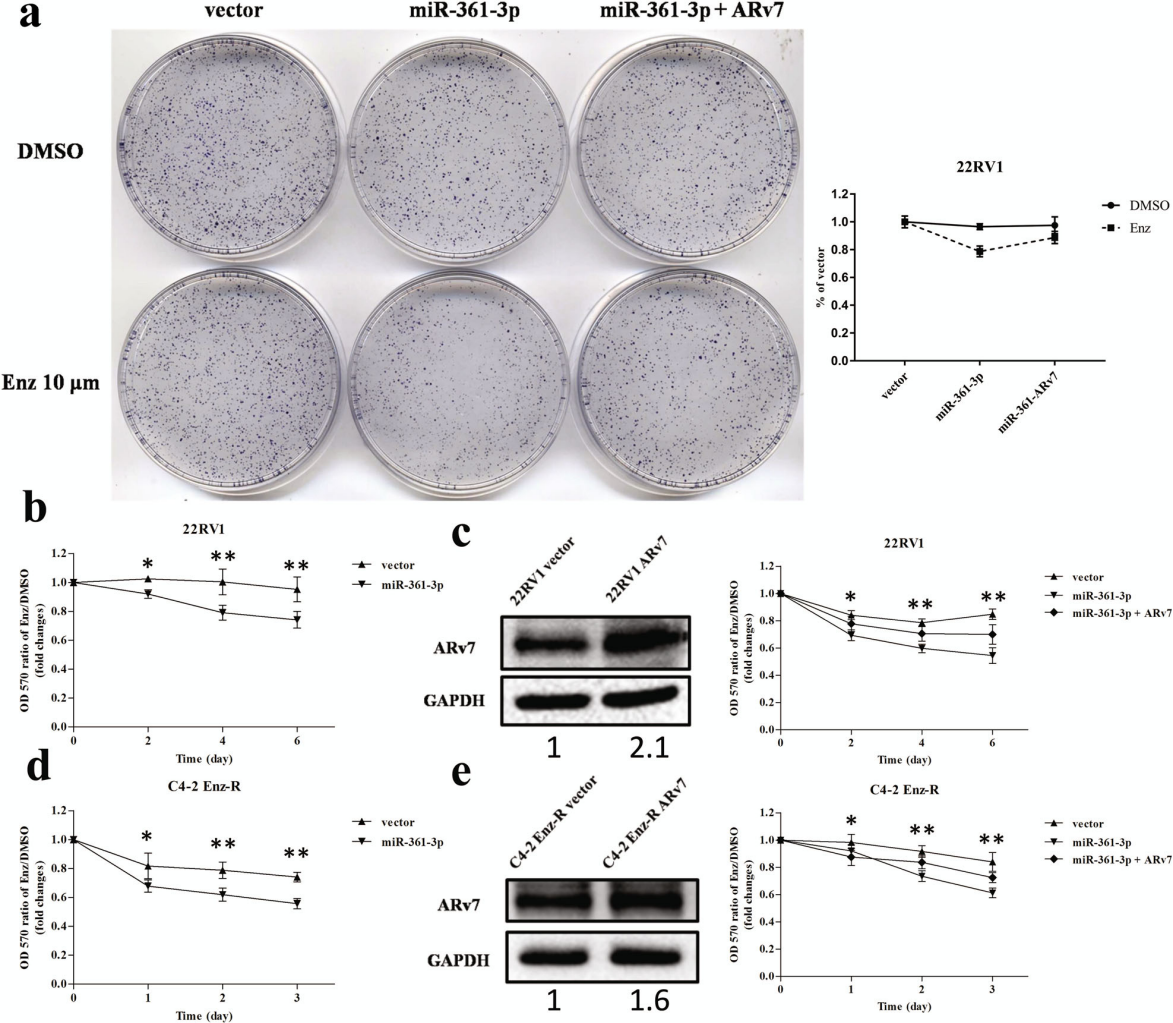
The miR-361-3p alters the Enz sensitivity of the CRPC cell growth through suppressing androgen receptor splicing variant 7 (ARv7). **a** Representative dishes and quantification results are shown for clongenic formation assays of CWR22Rv1 (22Rv1) cells. **b** Expressing miR-361-3p in 22Rv1 cells increases Enz sensitivity. **c** The miR-361-3p-suppressed Enz-mediated 22Rv1 cell growth is partially reversed after expressing the ARv7, left is western blot, right is MTT assay. **d** Expressing miR-361-3p decreases the cell growth in C4-2 Enz-R cells. **e** The miR-361-3p-suppressed Enz-mediated C4-2 Enz-R cell growth is partially reversed after expressing the ARv7, left is western blot, right is MTT assay. The final concentration of Enz in cell media is 10 μM. The values of the *Y* axis represent the ratios of cell vitality treated with Enz to DMSO (as control) in each group. Representative results are shown as mean ± SD. *P < 0.05, **P < 0.01.

Since recent clinical data indicated that a higher ARv7 expression might be linked to the development of Enz resistance in the CRPC patients receiving ADT–Enz^30,31^, we were interested to see if miR-361-3p-suppressed ARv7 may alter the Enz sensitivity. We first applied the clonogenic formation assay to examine Enz sensitivity during CWR22Rv1 cells’ growth. The results revealed that adding miR-361-3p led to increase the Enz sensitivity to better suppress the CRPC cell proliferation, and the results from the interruption approach further demonstrated that such increase can be reversed/blocked after adding exogenous ARv7 expression (Fig. 3a).

We then applied MTT cell growth/viability assays to further confirm the miR-361-3p effects for altering the Enz sensitivity in the CWR22Rv1 cells, and the results revealed that adding miR-361-3p could also lead to an increase in the Enz sensitivity to further suppress the cell growth in the CWR22Rv1 cells (Fig. 3b). Importantly, the results from an interruption approach also proved that such suppression of CWR22Rv1 cell growth could be reversed/blocked after adding the ARv7 (Fig. 3c). Similar results were also obtained when we replaced CWR22Rv1 cells with EnzR-C4-2 cells that were developed in vitro through long-term culturing in the presence of Enz, showing that adding miR-361-3p in the EnzR-C4-2 cells restored the sensitivity toward Enz (Fig. 3d), and this effect in cell growth could be reversed/blocked after adding exogenous ARv7 (Fig. 3e).

Together, the results from Fig. 3a–e suggest that miR-361-3p may function via binding specifically to the ARv7 3′UTR to suppress ARv7 protein expression to increase the Enz sensitivity to better suppress the CRPC cell growth.

### The miR-361-3p can also function via binding to MKNK2 3′UTR to suppress the MKNK2 expression to increase Enz sensitivity in the Enz-resistant cells

Interestingly, we found that adding ARv7 could only reverse partially the miR-361-3p-mediated Enz suppression of CRPC cell proliferation, suggesting that miR-361-3p might also regulate other signals to increase the Enz sensitivity. This is in agreement with recent studies showing that in addition to ARv7, other mechanisms, including expression of AR F876L mutant, or altering the glucocorticoid receptor signals, might also be able to contribute to the induction of Enz resistance in the CRPC cells^18,19,30,32–34^. Importantly, a recent study also indicated that miRNAs might function via binding to different genes’ 3′UTR to exert its unique biological function^27^.

**Fig. 4:**
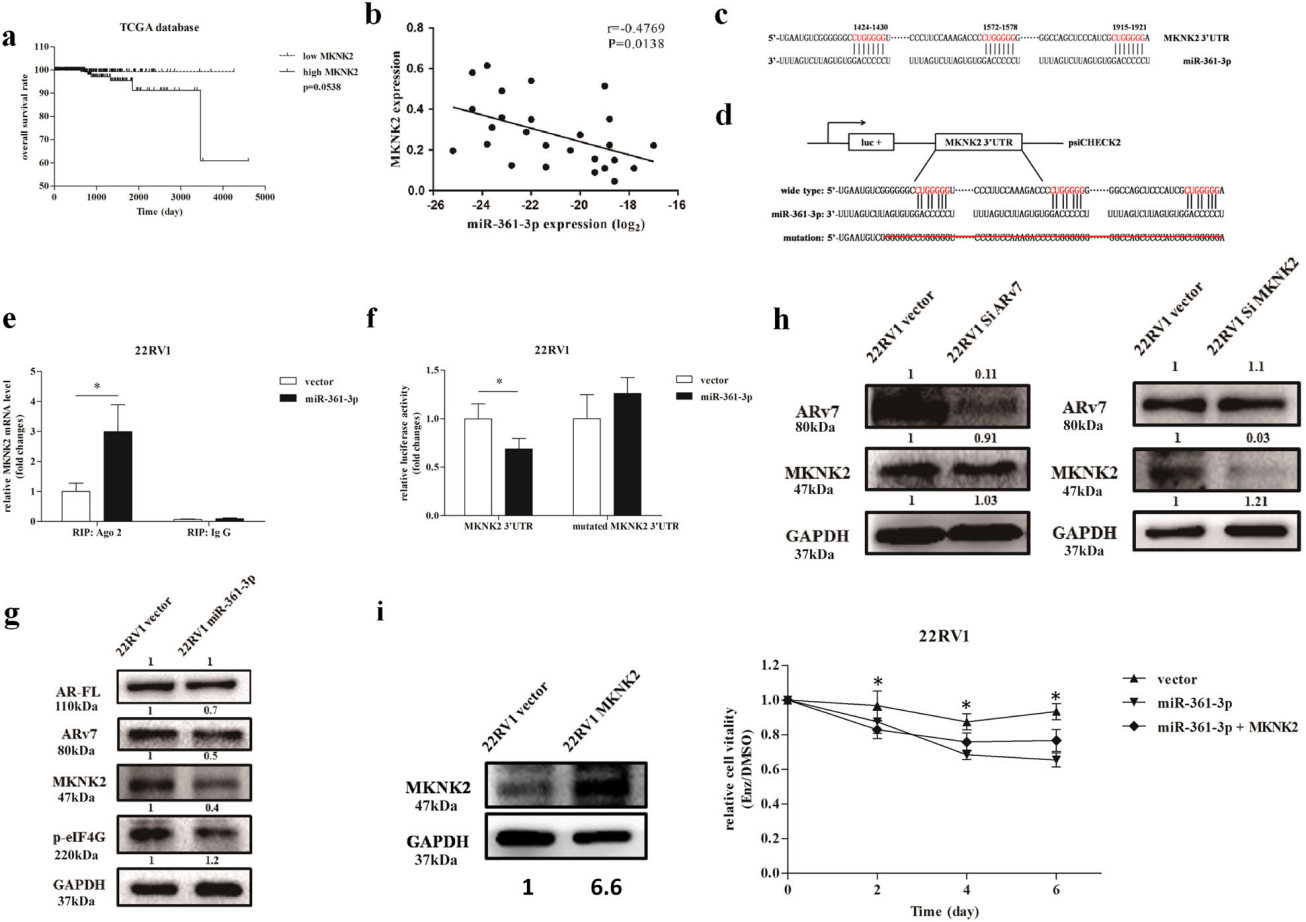
The miR-361-3p influences Enz sensitivity via targeting the MAP kinase-interacting serine/threonine kinase 2 (MKNK2) 3′UTR and altering MKNK2 expression. **a** TCGA database indicates that the higher MKNK2 level in human prostate cancer (PCa) tissues is correlated with lower overall survival rates. **b** The expression of MKNK2 is negatively correlated with the miR-361-3p level. **c** Sequence alliance of miR-361-3p to MKNK2 3′UTR. **d** The structure of luciferase reporter. **e** RNA immunoprecipitation (RIP) analysis of CWR22Rv1 (22Rv1) cells reveals recruitment of MKNK2 mRNA to microRNA ribonucleoprotein complex following immunoprecipitation against Ago2. IgG immunoprecipitation is used as a negative control. **f** Luciferase reporter assay reveals that adding miR-361-3p suppresses MKNK2 3′UTR luciferase activity in 22RV1 cells. The mutated MKNK2 3′UTR via deleting the miRNA- binding sites is used as control. **g** Western blot analysis shows that adding miR-361-3p suppresses MKNK2 and downstream gene p-eIF4G protein expression in CWR22Rv1 cells. **h** Suppression of androgen receptor splicing variant 7 (ARv7)/MKNK2 expression by shRNA could not change MKNK2 (left) or ARv7 (right) protein expressions. **i** Cell-proliferation assay shows that adding miR-361-3p increases Enz sensitivity in CWR22Rv1 cells, which can also be partially reversed by adding MKNK2. Representative results are shown as mean ± SD. *P < 0.05.

We then searched the online databases (TargetScan, MicroCosm, and miRDB) for other potential downstream target genes that could also be modulated by miR-361-3p. Interestingly, we found that miR-361-3p could also target MKNK2, whose function has been linked to the tumor growth via the classic translation/proliferation signals (MKNK2–eIF4G protein complex)^35–38^. Indeed, the results from the analysis of TCGA database (http://cancergenome.nih.gov) with the UCSC Cancer Genome Brower (https://genome-cancer.soe.ucsc.edu/) indicated that higher expression of MKNK2 in PCa is correlated with lower overall survival with a relaxed criterion of significance (*P* value = 0.0538) (Fig. 4a). The results from our collected human clinical sample also revealed that expression of MKNK2 is negatively correlated to the miR-361-3p at the mRNA level (Fig. 4b).

Sequence analysis revealed that the seed sequence of miR-361-3p could also bind to three sites of the MKNK2 3′UTR (Fig. 4c). The results from the RIP assay using anti-Ago2 antibody followed by adding miR-361-3p also revealed that MKNK2 mRNA could be specifically recruited to the miRNA ribonucleoprotein complex (Fig. 4e), and the results from the luciferase reporter assay using the MKNK2 3′UTR (structure in Fig. 4d) in CWR22RV1 cells also revealed that adding miR-361-3p in CWR22Rv1 cells resulted in an obvious reduction of luciferase activity, which was abolished after mutation of the MKNK2 3′UTR with deleting the miRNA-binding sites in the CWR22Rv1 cells (Fig. 4f).

Western blot analysis further confirmed that adding miR-361-3p in CWR22Rv1 cells decreased MKNK2 protein expression and its downstream effect, phosphorylation of eIF4G (p-eIF4G) (Fig. 4g).

Together, the results from Fig. 4a–g suggest that miR-361-3p could bind specifically to the MKNK2 3′UTR to suppress MKNK2–eIF4G signals. It is possible that suppression of MKNK2 will facilitate to increase the Enz sensitivity.

To determine whether miR-361-3p independently targets ARv7 and MKNK2, we used western blot assay and found that suppressing ARv7 via adding ARv7-shRNA failed to alter MKNK2 expression (Fig. 4h, left), while suppressing MKNK2 via adding MKNK2-shRNA also failed to alter the ARv7 expression in CWR22Rv1 cells (Fig. 4h, right). Importantly, an interruption approach via MTT assay also revealed that adding miR-361-3p could increase Enz sensitivity in CWR22Rv1 cells, while this sensitivity could be partially reversed with expression of MKNK2 (Fig. 4i).

Together, the results from Fig. 4a–i suggest that miR-361-3p could also function via binding to the MKNK2 3′UTR to suppress MKNK2 expression to increase the Enz sensitivity for better suppression of the Enz-resistant cell growth.

### Enz also functions via a feedback mechanism to influence the miR-361-3p expression in the CRPC cells

**Fig. 5:**
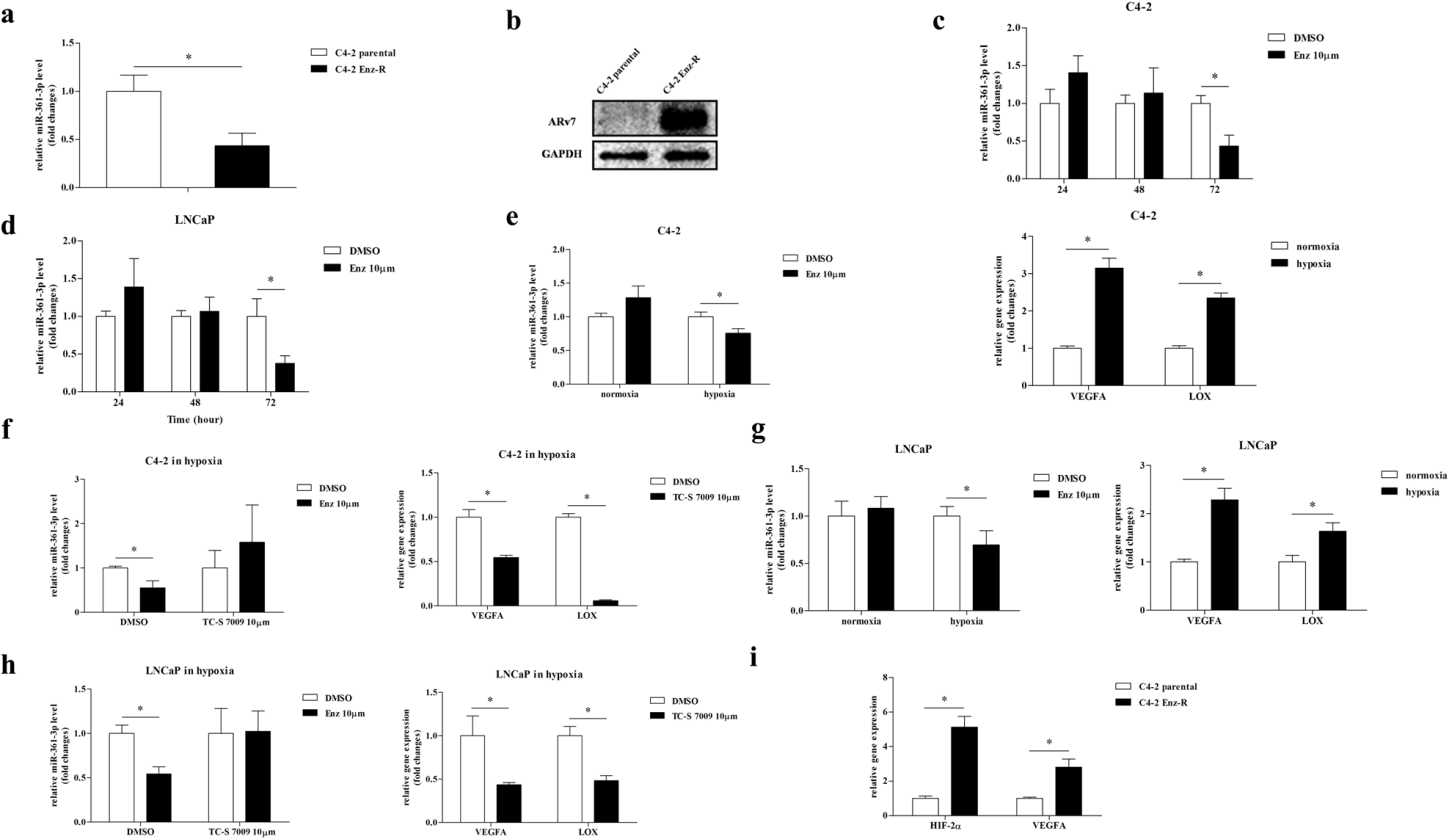
Mechanism dissection of how Enz influences miR-361-3p expression. **a** Quantitative real-time PCR analysis shows that miR-361-3p expression in C4-2 Enz-R cells is lower than in their parental Enz-sensitive C4-2 cells. **b** Western blot analysis shows that androgen receptor splicing variant 7 (ARv7) protein expression increased in C4-2 Enz-R cells compared to parental Enz-sensitive C4-2 cells. **c**, **d** Quantitative real-time PCR analysis shows that Enz decreases miR-361-3p expression in (**c**) C4-2 and (**d**) LNCaP parental cells after 72 h, but not at shorter times (24 or 48 h). **e** C4-2 cells treated with Enz for 24 h have decreased miR-361-3p expression under hypoxia (left); meanwhile, the expression of hypoxia-responsive genes is as expected (right). **f** Rescue assays show that decreased miR-361-3p expression in C4-2 cells could be reversed when hypoxia is antagonized by HIF-2α inhibitor TC-S 7009 (left). Expression of hypoxia-responsive genes is indeed reduced after treating with the inhibitor (right). **g** LNCaP cells treated with Enz for 24 h have decreased miR-361-3p expression under hypoxia (left); meanwhile, the expression of hypoxia-responsive genes is as expected (right). **h** Rescue assays show that decreased miR-361-3p expression in LNCaP cells could be reversed when hypoxia is antagonized by HIF-2α inhibitor TC-S 7009 (left). Expression of hypoxia-responsive genes is indeed reduced after treating with the inhibitor (right). **i** Hypoxia-responsive genes are increased in C4-2 Enz-R cells. Representative results are shown as mean ± SD. *P < 0.05.

Interestingly, the results from qPCR assay indicated that miR-361-3p expression in C4-2 Enz-R cells was lower than that in their Enz-sensitive parental C4-2 cells (Fig. 5a). This is in agreement with a higher ARv7 protein expression in C4-2 Enz-R cells (Fig. 5b), suggesting that Enz may also have a feedback mechanism to influence miR-361-3p expression in order to better control the ARv7 expression for the induction of Enz resistance in the CRPC cells.

To further dissect the molecular mechanisms of how Enz can alter the miR-361-3p expression, we examined the direct effect of Enz treatment on miR-361-3p expression with treatment-different durations. The results revealed that Enz could decrease miR-361-3p expression in C4-2 parental cells after 72 h, but not at a shorter interval (Fig. 5c), suggesting that Enz might function via indirect mechanisms to decrease miR-361-3p expression. Similar results were also obtained when we replaced C4-2 cells with LNCaP cells (Fig. 5d).

In an attempt to test whether Enz can more directly regulate miR-361-3p expression in a different cellular environment, we exposed the CRPC cells to hypoxia, a frequent occurrence in solid tumor progression. Indeed, we found that if C4-2 cells were exposed to hypoxia, then Enz could suppress the miR-361-3p expression even at 24 hr (Fig. 5e, left), and such suppression could be reversed after adding TC-S 7009 to inhibit HIF-2α, the key player of hypoxia signaling (Fig. 5f, left). Similar phenomena were also observed when we replaced C4-2 cells with LNCaP cells (Fig. 5g, h). The effects of hypoxia and anti-hypoxia signaling were further confirmed by other hypoxia-responsive genes, including VEGFA and LOX (Fig. 5e–h, right).

The detailed mechanism of how hypoxia can alter the Enz capacity to suppress the miR-361-3p expression remains unclear, although earlier studies indicated that ADT would induce hypoxia^39,40^, and a combination of Enz with hypoxia inhibitor therapy might result in synergistic inhibition of PCa cell growth^41^. We also found that the expression of HIF-2α and VEGFA is increased in C4-2 Enz-resistant cells compared with C4-2 parental cells (Fig. 5i), suggesting that Enz may be able to activate or enhance the hypoxia signal transduction.

Together, the results from Fig. 5a–i suggest that Enz can suppress the miR-361-3p expression in an indirect mechanism that probably involves altering the hypoxia signaling, which may then alter the miR-361-3p expression to modulate the expression of ARv7 and MKNK2 to alter the Enz sensitivity.

### Preclinical study using an in vivo mouse model using miR-361-3p to suppress the expression of ARv7 and MKNK2 to maximally increase the Enz sensitivity to better suppress the CRPC cell growth

To test the validity of all the above in vitro cell line studies in the preclinical study using an in vivo mouse model, and link to the potential clinical application, we then orthotopically xenografted the CWR22Rv1 cells (with stable expression of luciferase for IVIS image) into the anterior prostates of nude mice for four groups: (1) CWR22Rv1-Luc alone, (2) CWR22Rv1-Luc+ treated with Enz, (3) CWR22Rv1-Luc also expressing miR-361-3p, and (4) CWR22Rv1-Luc also expressing miR-361-3p+ treated with Enz.

**Fig. 6:**
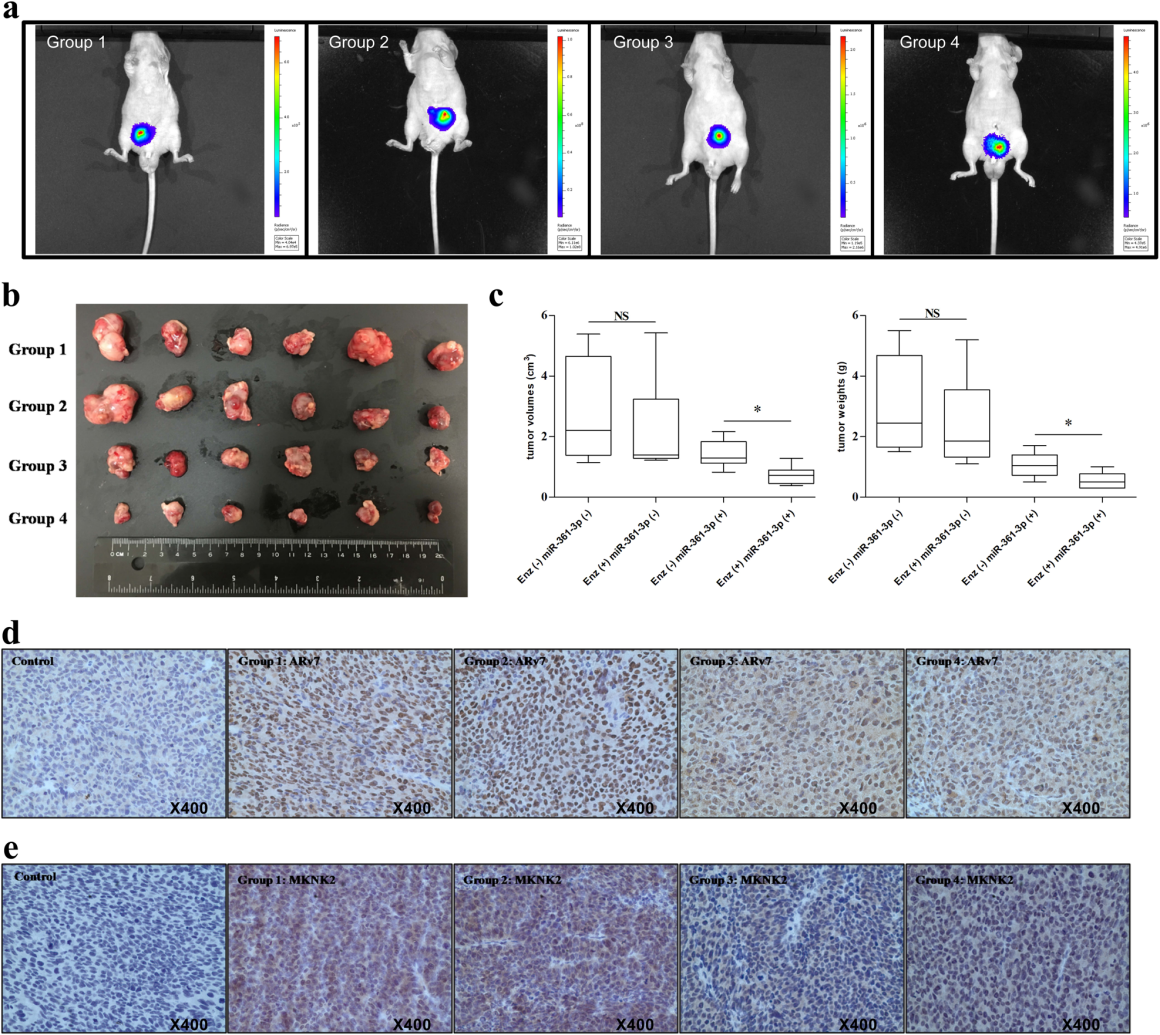
The miR-361-3p suppressed Enz-resistant prostate cancer (PCa) tumors in vivo. CWR22Rv1 cells with or without expression of miR-361-3p were labeled with luciferase and injected orthotopically into nude mice. After 1 week of implantation, each set of mice was randomly assigned into two experimental groups (treated with either DMSO or 35 mg/kg Enz by i.p. injection 3×/per week for 2 weeks). **a** One week after implantation, tumors were formed and visualized by IVIS image. Representative IVIS images of each group are shown. **b**, **c** Tumor sizes (**b**) and weights (**c**) in the four groups after sacrifice and tumor removal. ^*^P < 0.05. **d**, **e** IHC staining shows androgen receptor splicing variant 7 (ARv7) (**d**) and MAP kinase-interacting serine/threonine kinase 2 (MKNK2) (**e**) protein expression in four groups. We omitted the primary antibody in the control group. Representative IHC images of each group are shown. Magnification is ×400. Representative results are shown as mean ± SD. *P < 0.05, NS not significant.

Starting 1 week after orthotopic injection, tumor formation was visualized weekly by IVIS (Fig. 6a). The baseline tumor sizes of each group were roughly similar. In general, tumor growth in vivo matched cell growth in vitro showing that Enz treatment for 2 weeks suppressed the CWR22Rv1-Luc tumor growth (group 2 vs group 1; 21% and 20% decrease in tumor size and weight, respectively) compared to CWR22Rv1-Luc tumor growth (Fig. 6b, c), although without a statistical significance. Expressing miR-361-3p alone also suppressed the CWR22Rv1-Luc tumor growth (group 3 vs group 1; 49% and 64% decrease in tumor size and weight, respectively, Fig. 6b, c). Importantly, combining Enz and miR-361-3p led to the most significant suppression effects (group 4 vs group 3; 49% and 48% decrease in tumor size and weight, respectively, Fig. 6b, c).

The results from immunohistochemistry (IHC) staining also confirmed that the in vitro cell lines showing mice with combined miR-361-3p with Enz treatment (in group 4) had significantly decreased ARv7 (Fig. 6d) and MKNK2 (Fig. 6e) protein expression.

Together, the results from preclinical studies using the in vivo mouse model (Fig. 6a, e) demonstrated that increased miR-361-3p expression may lead to an increase in Enz sensitivity to better suppress the Enz-resistant CRPC cell growth via suppressing the expression of ARv7 and MKNK2.

## Discussion

The miR-361-3p has been identified as a tumor suppressor whose expression is decreased in multiple recurrent human cancers^28^, including in PCa showing much lower expression in the PCa sample compared with benign prostate hyperplasia tissues^29^. Another study also reported that the expression of miR-361-5p, another miRNA product from the same parental miRNA-361 precursor, was also lower in CRPC than in androgen-dependent prostate cancer (ADPC), suggesting that miR-361-5p and miR-361-3p may play negative roles in the progression of ADPC to CRPC^42^.

Here, we demonstrated the tumor-suppressor role of miR-361-3p that may contribute to the development of Enz resistance in multiple CRPC cells and in vivo mouse models. We found that miR-361-3p has the specific target sequence in the ARv7 3′UTR, but not in the 3′UTR of the fAR (Fig. 2a). This miRNA-target interaction led to suppressing the ARv7 protein expression to increase the Enz sensitivity in vitro and in vivo.

Importantly, the results from our studies also strongly suggest that the ARv7 is not the sole player to contribute to the development of Enz resistance. A recent study also demonstrated that Enz sensitivity of PCa cells can be regulated by ARv7 along with EZH2 and Src^43^. In our study, MKNK2 was demonstrated to play an important role in Enz resistance. This finding is clinically significant since MKNK2, or other molecules, may be the reason why some ARv7-negative patients had a poor response to the new next-generation AR-targeting therapies (abiraterone or Enz)^30^. Furthermore, our study highlighted the significance of regulating ARv7 expression at multiple levels. It is very likely that ARv7 protein in CRPC is ultimately responsible for the efficacy of Enz therapy, yet mRNA expression of ARv7 may not be predictive of its function as it can be regulated by post-transcriptional regulation such as with miR-361-3p. Indeed, a recent study indicated that detection of ARv7 mRNA in patient blood did not predict the reduction of serum PSA in patients with CRPC following abiraterone or Enz administration^44^, consistent with the view that ARv7 mRNA might be regulated by miRNAs, which ultimately determine the ARv7 protein expression and treatment efficacy. It is conceivable that other post-transcriptional and post-translational regulations might also exist to influence ADT–Enz sensitivity in CRPC.

Our study also dissected the mechanism of Enz-influenced miR-361-3p expression, and found that Enz could suppress the miR-361-3p level, which may not involve the transcriptional regulation since such suppression effect occurred after Enz treatment for a relatively long time. Instead, we found that such regulation might involve the alteration of the hypoxia signaling, and we demonstrated that the hypoxia signals could be activated in Enz-resistant PCa cells. These findings suggest that Enz treatment would induce the cellular hypoxia microenvironment and activation of hypoxia signaling, which in turn could contribute to the development of Enz resistance in the CRPC cells. Indeed, Fernandez et al.^41^ have shown that dual targeting of the AR and hypoxia signals might lead to synergistic suppression effects on Enz-resistant cell growth^38^.

In conclusion, we find that miR-361-3p can specifically bind to the 3′UTRs of ARv7 and MKNK2 to maximally suppress their expression to increase the Enz sensitivity. Combining miR-361-3p with ADT–Enz therapy may be developed as a novel therapy to better suppress CRPC at the Enz-resistant stage.
